# Local Immune Changes in Early Stages of Inflammation and Carcinogenesis Correlate with the Collagen Scaffold Changes of the Colon Mucosa

**DOI:** 10.3390/cancers13102463

**Published:** 2021-05-18

**Authors:** Fabián Čaja, Dmitry Stakheev, Oleksandr Chernyavskiy, Lucie Kubinová, Jiří Křížan, Jiří Dvořák, Pavel Rossmann, Renata Štěpánková, Peter Makovický, Pavol Makovický, Veronika Vymetalková, Pavel Souček, Pavel Vodička, L’udmila Vodičková, Miroslav Levý, Luca E. Vannucci

**Affiliations:** 1 Institute of Microbiology of the CAS, v. v. i., Vídeňská 1083, 142 20 Prague 4, Czech Republic; caja@biomed.cas.cz (F.Č.); stakheev@biomed.cas.cz (D.S.); jiri.krizan@seznam.cz (J.K.); Jiri.Dvorak@uhkt.cz (J.D.); immuno@biomed.cas.cz (P.R.); Stepankova.Renata@seznam.cz (R.Š.); 2 Faculty of Science, Charles University, Albertov 6, 128 00 Praha 2, Czech Republic; 3 Institute of Physiology of the CAS, v. v. i., Vídeňská 1083, 142 20 Prague 4, Czech Republic; Oleksandr.Chernyavskiy@monach.edu (O.C.); Lucie.Kubinova@fgu.cas.cz (L.K.); 4 Institute of Experimental Oncology, Biomedical Research Center of the Slovak Academy of Sciences Dubravska 9, 845 05 Bratislava, Slovakia; peter.makovicky@savba.sk; 5 Pedagogical Faculty, Selye Janos University, Bratislavská cesta 3322, 945 01 Komárno, Slovakia; makovickyp@ujs.sk; 6 Institute of Experimental Medicine of the CAS, v. v. i., Vídeňská 1083, 142 20 Prague 4, Czech Republic; veronika.vymetalkova@iem.cas.cz (V.V.); pvodicka@biomed.cas.cz (P.V.); ludmila.vodickova@iem.cas.cz (L.V.); 7 Biomedical Center at Medical Faculty of Charles University, alej Svobody 1655/76, 323 00 Plzeň—Severní Předměstí, Czech Republic; 8 Institute of Biology and Medical Genetics, Charles University, Purkyně Institute, Albertov 4, 128 00 Prague 2, Czech Republic; 9 Centre of Toxicology and Health Safety, The National Institute of Public Health, Šrobárova 49/48, 100 00 Praha 10, Czech Republic; pavel.soucek@szu.cz; 10 Thomayer University Hospital, 1st Medical Faculty, Charles University, Vídeňská 800, 140 59 Praha 4, Czech Republic; miroslav.levy@ftn.cz

**Keywords:** colorectal cancer, DSS-induced colitis, chronic inflammation, collagen, tissue scaffold, AOM, tumour niche, IL-6

## Abstract

**Simple Summary:**

Chronic colitis and colon cancer develop for alteration of the mucosa homeostatic regulation, also involving TGF-β1. Dextran sulphate sodium (DSS)-induced colitis and azoxymethane (AOM)-induced colorectal carcinogenesis animal models allow for the investigation of the pathological evolution steps. Since chronic inflammation is a common factor, we aimed to explore in rat models the colon mucosa immunological and structural conditions at one month after the end of the inductions, a transition period between acute effects and established lesions. We found, in comparison to healthy controls, downregulation of inflammatory cytokines (except IL-6) and of TGF-β1. At the same time, the collagen scaffold was significantly remodelled in both groups. We conclude that the pro-inflammatory cytokines, in front of a downregulated TGF-β1, sustained a smouldering inflammation with structural changes preparing the niche of both pathologies (ulcerative colitis with fibrosis; tumour). The collagen scaffold changes pointing to an unnoticed inflammation may be suggested as a possible pre-neoplastic condition marker.

**Abstract:**

Continuous activation of the immune system inside a tissue can lead to remodelling of the tissue structure and creation of a specific microenvironment, such as during the tumour development. Chronic inflammation is a central player in stimulating changes that alter the tissue stroma and can lead to fibrotic evolution. In the colon mucosa, regulatory mechanisms, including TGF-β1, avoid damaging inflammation in front of the continuous challenge by the intestinal microbiome. Inducing either DSS colitis or AOM colorectal carcinogenesis in AVN-Wistar rats, we evaluated at one month after the end of each treatment whether immunological changes and remodelling of the collagen scaffold were already in development. At this time point, we found in both models a general downregulation of pro-inflammatory cytokines and even of TGF-β1, but not of IL-6. Moreover, we demonstrated by multi-photon microscopy the simultaneously presence of pro-fibrotic remodelling of the collagen scaffold, with measurable changes in comparison to the control mucosa. The scaffold was significantly modified depending on the type of induced stimulation. These results suggest that at one month after the end of the DSS or AOM inductions, a smouldering inflammation is present in both induced conditions, since the pro-inflammatory cytokines still exceed, in proportion, the local homeostatic regulation of which TGF-β1 is a part (inflammatory threshold). Such an inflammation appears sufficient to sustain remodelling of the collagen scaffold that may be taken as a possible pathological marker for revealing pre-neoplastic inflammation.

## 1. Introduction

In the last 20 years, the concept of tumour microenvironment has continuously developed, becoming an effective working model. It can permit the proper reconstruction of the relationships between tissue components and transformed cells and allows for a better understanding of the events and mechanisms of the tumour evolution. This is important to properly design and elaborate anticancer interventions and more appropriate clinical approaches [1,2,3,4]. The tumour microenvironment consists in the co-presence of transformed cells together with normal cells of the original tissue, stromal cells and their products (e.g., collagen), neovascularisation, nerves, lymphatics, and immune cells and their products. The immune cells are an important component of this network, and inflammation is recognised to have a relevant part in the carcinogenesis process and tumour development. The activation of innate immunity elicited by tissue stress signals under the pressure of the developing tumour clone stimulates a local inflammatory Th1 response activating natural killer (NK) cells and macrophages and further involving the adaptive immunity [1,2,5]. The failure of the anticancer response is associated with the shift of immunity towards an immune-suppressive and tissue remodelling Th2 response and the accrual of T regulatory (Treg) cells, M2 macrophages, and of myeloid derived suppressor cells (MDSC) that deliver in the microenvironment immune suppressive molecules (e.g., TGF-β, IL-10, arginase-1) [6]. Stromal structures are also involved in the process [7,8]. The interactions between immunity and fibroblasts shaping the chronically inflamed tissues, such as in the inflammatory bowel diseases (IBD), have progressively attracted the interest of researchers for their importance both in neoplastic and non-neoplastic pathologies and for the active role of fibroblasts in these contests. A fibrotic reaction with increased collagen deposition is often found in various types of tumours [7,8,9]. In relation to this, the IBD as well as the colorectal carcinoma (CRC) offer a particularly valuable model to study and compare the mechanisms and effects of inflammation, since these different pathologies affect the same structures in the same organ. Moreover, the bowel mucosa is a composite structure and can be considered as the most important immunological organ in the body due to its rich and diffused mucosa-associated lymphocyte population. For its particular characteristics, including the continuous interaction between mucosal immunity and commensal microflora, the bowel mucosa can be considered an excellent model for evaluating inflammation and its regulation [10,11,12,13,14].

Chronic inflammation resembling IBD or cancer-promoting conditions can be easily induced in mouse and rat models using dextran sodium sulphate (DSS) for IBD-like colitis or azoxymethane (AOM) for colorectal carcinogenesis [15,16,17,18].

DSS is a branched poly-α-D-glucoside in a sodium sulphate formulation that permits solubility and stability in water, and, at 30–50 kDa MW, has colitogenic effect. It induces a strong intestinal inflammation when orally administered in drinking water to experimental animals, for an established period, at the concentration of 1–5%, depending on the chosen experimental animal model. It produces the disruption of epithelial barrier function, increasing permeability and favouring trans-mucosal penetration of bacteria [15,18]. They can interact with bacterial molecular pattern Toll-like receptor (TLR-2, TLR-4), activating pro-inflammatory cytokine production, mainly by the macrophages. Release of alarmins may derive by damage of colonocytes due to nano-lipocomplexes of DSS and medium-chain-length fatty acids (MCFAs) that can fuse with the cell membrane producing cytotoxic effects [19]. The consequent acute inflammation leads to further mucosal cell damage, infiltration of activated immune cells into the colon epithelia, and tissue alterations with also loss of mucosa continuity (erosions, ulcers). DSS-related acute tissue injury can progress to IBD-like chronic inflammation if repeated cycles of the polysaccharide are applied. In this process, IL-6 plays an important role, even in the progression to fibrosis [18,20,21,22,23,24,25].

AOM, the most commonly used colorectal carcinogen, is an active derivative of 1,2-dimethylhydrazine. Cytochrome P450 2E1 (CYP2E1) transforms AOM in methylazoxymethanol that induces O6-methylguanine adducts in the DNA, leading to G→A transitions [16]. Mutations in the KRAS gene with activation of the PI3K/Akt pathway, β-catenin mutations, accumulation and decrease of TGF-β active form, and inhibition of TGF-β pathway are all mechanisms involved in the AOM colorectal carcinogenesis process [26]. Moreover, increased TLR-4 was also found to be associated with the AOM-induced tumorigenesis [27]. AOM induces formation of aberrant crypt foci in the colon tissue with following sequence from adenoma to carcinoma after repeated administrations. Its activity is enhanced by cancer promoters such as secondary biliary acids and induced inflammation [28,29,30].

Even though DSS colitis and AOM carcinogenesis models are widely applied, studies are still ongoing as to their mechanisms of action and effects at cellular and immunological levels. A lack of information remains about the documentation of biological and immunological features in the transition period between the acute and the fully established outcomes of each type of induction.

**Figure 1:**
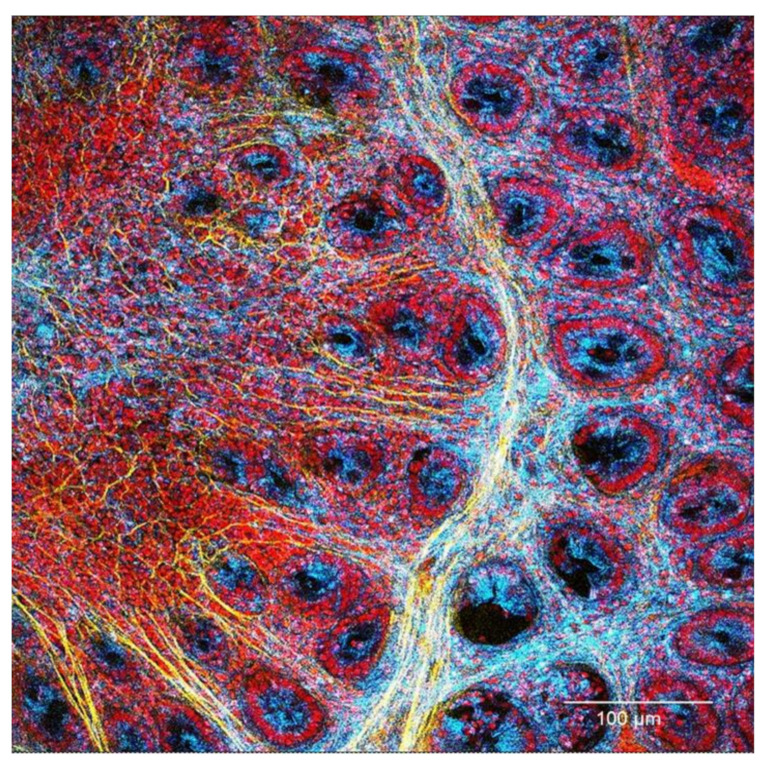
Lymphatic follicle in the colon mucosa at one month after the end of DSS colitis induction. The collagen structure surrounding the follicle (yellow) appears thickened. Increased signal of the connective tissue is present also in the inter-cryptal spaces (lower right in the figure, light blue and yellow colours). Merging of Draq5 nuclear staining (red, confocal microscopy), mucosa (blue, reflectance confocal microscopy—RCM), and collagen (yellow, second harmonic generation—SHG by multi-photon confocal microscopy); objective HC PLAPO 20×/0.70 lMM CS with water immersion.

Therefore, in this study, we selectively focused on the events at one month from the end of DSS and AOM induction in rat model in order to describe possible structural and immunological changes in the colon mucosa characterising this transition period. The results indicated that evident collagen structure remodelling and local immune changes are already present in both models at this time point (Figure 1). The immune network appeared deregulated and even partially suppressed, but with relative pro-inflammatory cytokines in comparison to the regulatory TGF-β1 levels. These new results indicate that the modification of an “inflammatory threshold” maintaining the immunological homeostasis in the colon mucosa may permit the persistence and resilience of inflammation even under apparently controlled conditions (as a latent inflammation).

## 2. Materials and Methods

### 2.1. Experimental Animals and Design of the Experiment

Male inbred AVN-Wistar (F89) rats, 150 g medium body weight, were purchased from and housed in the Institute of Physiology of the CAS, v. v. i. in Prague, Czech Republic [31,32,33]. The rats were reared under standard conditions, with 12 h light/darkness cycle and without food and water restrictions. All experiments were designed to include minimally 5 animals per group in each used method. The housing conditions and the experimental procedures respected the rules of the European Convention for the Care and Use of Laboratory Animals (2010/63/EU) as approved by the Czech Animal Care and Use Committee and by the Institutional Ethics Committee.

Dextran sodium sulphate (DSS, MW = 36,000–50,000 Da, cat. no. 160110 lot. no. 5237K, MP Biomedicals, Irvine, CA, USA) was used to induce chronic colitis, and azoxymethane (AOM, Sigma-Aldrich, St. Louis, MO, USA) was used to induce colon carcinogenesis.

**Figure 2:**
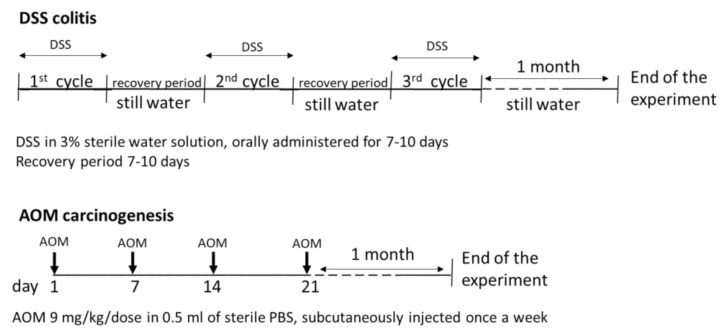
Design of the experiment.

Colon inflammation was induced by 3% DSS sterilised water solution, supplied to the rats by drinking ad libitum during three treatment cycles of 7–10 days each. DSS solution administration was interrupted at the appearance of acute symptoms of colitis (anal oedema, diarrhoea, and/or rectal prolapse or bleeding) and substituted with drinking water ad libitum during the recovery period of ~1 week before the following cycle (Figure 2). Animal scoring for disease activity index (DAI) was performed at the end of each cycle and at the end of the experiment, i.e. at one month after completing the DSS treatment. The scoring system was changes in weight loss (score: 0, none; 1, 1–5%; 2, 5–10%; 3, 10–20%; and 4, >20%), stool consistency (score: 0, formed; 1, mildly soft; 2, very soft; and 3, watery stool), and bleeding (score: 0, normal; 1, brown; 2, reddish; and 3, bloody).

Carcinogenesis was induced by subcutaneous injection of 4 doses of AOM, 1 dose weekly, 9 mg of AOM/kg/dose in 0.5 mL of sterile PBS (Figure 2). All rats were sacrificed one month after the last cycle of treatment by total bleed under general anaesthesia (0.8% chloralium hydrate). The necropsy was performed with gross examination of internal organs. Spleen and mesenteric lymph nodes (MLN) were harvested. The large bowel was isolated, longitudinally opened, and washed from stools with cold saline, and the total length was measured. Before the sampling of the mucosa, samples of fresh colon tissue were taken, partly for direct confocal microscopic analysis and partly to be fixed in 10% buffered pH 6.9 formalin for standard histology. Samples of colonic mucosa (right and left colon) were collected by gently scraping it with a sterile glass slide. Part of the obtained mucosa was immediately snap-frozen in liquid nitrogen and stored at −80 °C for further analyses, part was stored in RNA later (Life Technologies, Carlsbad, CA, USA) and at −80 °C, if not immediately processed, to be used for real-time polymerase chain reaction (RT-PCR) analysis (Life Technologies, Carlsbad, CA, USA). The fresh colon samples were directly transported to the confocal microscope for immediate evaluation. The specimens for histology were sent to the pathologist.

### 2.2. Histopathological and Histochemical Evaluation

All samples were fixed with a 10% buffered pH 6.9 formalin solution, preserved for 24 h and then embedded in paraffin, processed according to standard histological methods. Three to five micrometre-thick slices were cut from each sample and were stained with haematoxylin–eosin (HE). For histochemistry, other serial sections were cut from each sample and stained with picrosirius staining for collagen type I and reticulin using Sirius Red kit (DiaPath, Martinengo, Italy) according to the manufacturer’s protocol. The final preparations were evaluated and described using an Olympus Provis BX50 microscope (Olympus, Tokyo, Japan). Images were acquired using an Olympus DP 70 Photo camera with QuickPhoto Micro 23 program (Olympus, Tokyo, Japan). Samples were independently evaluated by two expert pathologists (P.R. and P.M.).

#### Confocal Microscopy Analysis

All observations were performed with a Leica TCSSP2 AOBS laser scanning confocal microscope based on Leica DM IRE2 inverted stand and equipped with an argon laser. Images were acquired using a HC PLAPO 20x/0.70 lMM CS multi-immersion objective with water immersion (WD 0.250 mm). For higher magnification, we used either digital zoom of areas of interest or, in some cases, a HCX PL APO 63x/1.20 W Corr water-immersion objective (WD 0.220 mm). For visualisation of reflection (reflectance confocal mode—RCM) from all tissues under study, we applied three excitation wavelengths of 488, 514, and 633 nm with overlaying acquired images, resulting in a histology-like final image. The reflected signal detection was obtained in up to three corresponding channels, using detection wavelength ranges of 478–498, 504–524, or 623–643 nm, respectively. Each of the three different excitation wavelengths applied in reflection mode was valuable and brought specific information due to its specific scattering characteristics. The second harmonic generation (SHG) technique was used for investigation of collagen fibre structures. The autofluorescence of non-stained tissues was detected by 2-photon excitation microscopy at the wavelength of 860 nm, which implied expected detection of SHG signal at the wavelength of 430 nm, as previously described by Chernyavskiy et al. [34]. To confirm the detection of SHG signal, we checked if the signal disappeared when the excitation wavelength was changed to 800 nm, and then re-appeared after setting up a band pass filter for 400 nm. Image J software^®^ (NIH, MA, USA) was used for analysis of the intensity of each pixel in a given microscopic field by various parameters: integrated density (ID), inter-cryptal space, and distance between centres of the crypts and skewness (symmetry and regularity of fibre distribution). For each examined image, analysis was performed of almost 5 representative fields per sample. For each colon, a mean of 5 samples was taken.

### 2.3. Isolation of RNA and RT-PCR Analysis

The colon mucosa samples were stored in RNA later (Life Technologies, Carlsbad, CA, USA) at −80 °C until further analysis. Total RNA was isolated from tissues using TRIZOL reagent according to the manufacturer’s protocol (Life Technologies, Carlsbad, CA, USA). Purity of RNA was measured using a UV–VIS NanoPhotometer^®^ P300 (Implen, München, Germany). Only samples with OD260/280 between 1.8 and 2.0 were used for RT-PCR analysis. Quality of RNA was evaluated by denaturing agarose gel electrophoresis (Bio-Rad, Hercules, CA, USA). DNAse I-treated total 2 µg of RNA were reverse-transcribed using Oligo(dT)20 primer and Superscript III Reverse Transcriptase (Life Technologies, Carlsbad, CA, USA), and then we used a real-time PCR (RT-PCR) reaction to determine the changes in the mRNA level. The level of expression of selected genes was evaluated by using the iCyclerTM iQ5TM RT-PCR detection system (Bio-Rad, Hercules, CA, USA). The list of studied genes and used primers is found in Table 1. The PCR reaction was performed in a volume of 25 μL, containing 4 μL of cDNA (10 ng/μL), 12.5 μL of SYBR^®^ Green Supermix (Bio-Rad, Hercules, CA, USA), and 500 nM of each primer. Non-template controls were included in all experiments. The setup of RT-PCR reaction was as follows: 3 min at 95 °C, 40 cycles of 94 °C for 30 s, 59 °C for 40 s, and 72 °C for 70 s. The temperature was then gradually increased to 95 °C to obtain the melting curves of the amplified fragments. The efficiency of primer pairs was estimated around 98%. All reference genes (Gadph, Pgk I, Ppia, Polr2A, Surf1, Tbp) were validated using Bestkeeper, Normfinder, and RefFinder algorithms, and Ppia gene was chosen as the most stable in the defined reaction conditions [35,36]. Reverse transcription was also performed without Superscript III for all samples (No-RT-control) and successful removal of gDNA was confirmed by PCR. Ppia gene was used in the assay. No PCR product was detected in all samples. Changes in the gene expression were shown as the normalised expression in treated rats compared to controls. Two independent experiments were performed. All parameters were measured in duplicates in each experiment. Changes in gene expression were calculated according to the 2-ΔΔCT (Livak) method [37]. Representative melting curves of the evaluated genes are reported in Supplementary Materials, Figure S1. 

### 2.4. Isolation of Proteins

Protein lysates were prepared according to the following protocol: each sample was placed in 400 µL of tissue extraction buffer T-PER (Thermo Scientific, Waltham, MA, USA). Protease inhibitors cOmplete Mini and Pierce Protease Inhibitor were used to protect samples from spontaneous degradation at 1 tablet/10 mL of T-PER buffer (Roche Diagnostics GmbH, Mannheim, Germany; Thermo Scientific, Waltham, MA, USA). Tissue samples were homogenised by FastPrep^®^-24 Instrument (MP Biomedicals, Santa Ana, CA, USA). Lysing Matrix D ceramic balls were added to each vial to help homogenise the samples (MP Biomedicals, Santa Ana, CA, USA). The procedure was followed by two cycles of 60-s homogenisation at 6.5 m/s speed. All samples were held in cold conditions on ice. Then, the vials were centrifuged (HERMLE Z 383 K refrigerated centrifuge and HERMLE 220.87 V03/V04 rotor, HERMLE Labortechnik GmbH, Wehingen, Germany) at 4 °C and 15,000× *g* rpm for 15 min, and supernatants were collected and stored in −80 °C for further analysis.

### 2.5. ELISA Assay

All protein samples were analysed by sandwich ELISA method. The experiments were performed according to the manufacturer’s protocols. The protein lysates, stored at −80° C, were used in a reaction volume 100 µL/well. All samples were diluted to the same concentration and measured in a doublet; concentration of protein lysates was measured by UV–VIS NanoPhotometer^®^ P300 (Implen, München, Germany). The results were calculated according to a standard curve. The following ELISA kits were used: rat IL-1α Matched Antibody Pairs kit (eBioscience, San Diego, CA, USA), rat IL-6 DuoSet ELISA Development kit (R&D Systems, Minneapolis, MN, USA), TGF-β CytoSet (Thermo Scientific, Waltham, MA, USA), rat IFN-γ ELISA Development kit (PeproTech, Rocky Hill, NJ, USA). ELISA plates were measured using Multiskan Ascent ELISA plate reader (Thermo Scientific, Waltham, MA, USA).

### 2.6. Statistical Evaluation

The results are expressed as means ± standard error of mean (S.E.M.). Differences in distributed variables between groups were compared by one-way ANOVA and subsequent Kruskal–Wallis test using GraphPad Prism™. Statistical significance was considered for *p* < 0.05. Data related to the imaging analysis, ELISA and PCR evaluations are summarized in the Table S1.

## 3. Results

### 3.1. Evaluation of Changes in the Colon Mucosa Scaffold

AOM-treated animals did not present any alteration in functions and behaviour at any time during the entire experiment. A disease activity index (DAI) was assessed for the DSS-treated rats at the end of each cycle of treatment and at the end of the experiment (1 month after the end of DSS induction). Weight loss was mild (DAI score 1) during all experiments, occurring in 25% of animals after the first and second cycles, 8.33% after the third cycle, and 27.27% after one month. At the same observation time, bleeding was evident (DAI score 2–3) in 75%, 83.83%, and 58.33% of rats, respectively, while being absent after one month from the induction. Soft stools and, occasionally, fluid stools (DAI score 2–3) were observed after the first and second cycle, while only grade 2 was observed after the third cycle (with incidences of 75%, 100%, and 91.66%, respectively). After one month, about 63% of the animals still presented softened stools (DAI score 1–2), confirming the established chronic colitis.

All animals were sacrificed at one month after the end of the respective induction protocol. At the autopsy, the gross evaluation of the abdominal and thoracic organs did not show any notable modification or lesion. The colon mucosa, under both conditions, did not present macroscopically visible alterations, and only the colon of DSS animals appeared shortened in comparison to control rats.

**Figure 3:**
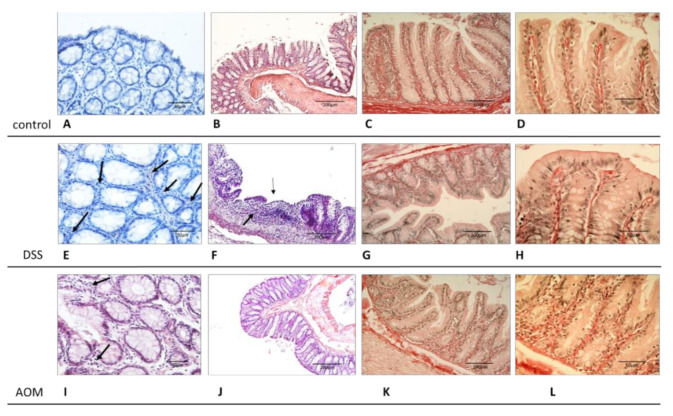
Histology of the colon mucosa from healthy, DSS-, and AOM-treated rats. The histological evaluation showed modification in the regular disposition and homogeneous shape of the crypts associated with increased cellular inter-cryptal infiltrate after DSS and AOM treatments in comparison to the control (**A**,**E**,**I**). The DSS-treated group showed evidence of colitis with parvicellular infiltrate ((**E**), arrows, and (**F**)); reduction of the crypt length; and, sporadically, little ulcerations (**F**). Mild changes in the crypts shape and cell infiltrate (**I**, arrows) resulted in the mucosa after AOM induction (**I**–**L**). The collagen structures appeared enhanced after each type of treatment (picrosirius stain, red; (**D**,**H**,**L**)). (**A**–**D**) healthy rats; (**E**–**H**) DSS-treated rats; (**I**–**L**) AOM-treated rats; (**A**,**B**,**E**,**F**,**I**,**J**) haematoxylin–eosin staining; (**C**,**D**,**G**,**H**,**K**,**L**) picrosirius staining; (**A**,**E**,**I**) and (**D**,**H**,**L**) 40× magnification; (**B**,**F**,**J**) 10× magnification; (**C**,**G**,**K**) 20× magnification.

**Figure 4:**
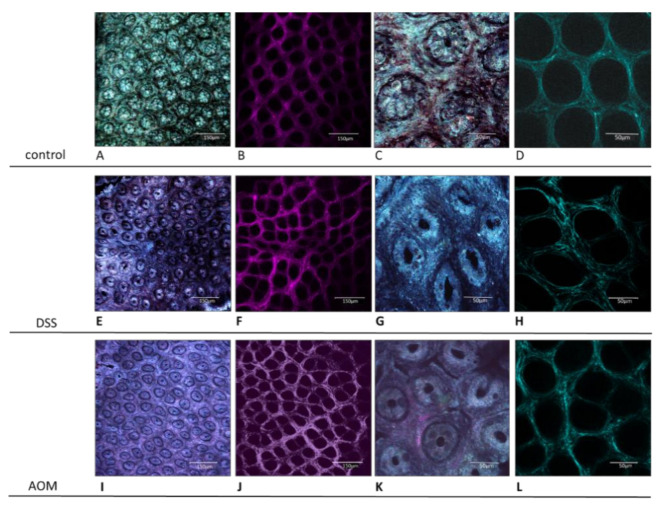
Healthy and treated rat colon mucosa evaluation by confocal microscopy. Confocal microscopy revealed more precisely the structural changes in the colon tissue related to the cryptal symmetry (**A**,**E**,**I**) and shape (**C**,**G**,**K**) and the collagen architecture (**B**,**D**,**F**,**H**,**J**,**L**). More irregular and dense organisation of collagen structures induced by both DSS (**F**,**H**) and AOM (**J**,**L**) in comparison with control animals (**B**,**D**). Inter-cryptal spaces were less regular and wider (**A**,**E**,**I**), and the shape of the crypts (**C**,**G**,**K**) resulted in being especially modified by the DSS treatment (**G**). (**A**–**D**) healthy rats; (**E–H**) DSS-treated rats; (**I**–**L**) AOM-treated rats; (**A**,**C**,**E**,**G**,**I**,**K**) reflectance confocal mode, 1-photon confocal microscopy; (**B**,**D**,**F**,**H**,**J**,**L**) second harmonic generation, multi-photon confocal microscopy. (**A**,**B**,**E**,**F**,**I**,**J**) HC PLAPO 20×/0.70 lMM CS objective with water immersion; (**C**,**D**,**G**,**H**,**K**,**L**) HCX PL APO 63×/1.20 W Corr water-immersion objective (WD 0.220 mm).

Samples from the harvested colons were prepared for histological evaluations and for confocal microscopy analysis. Sections of paraffin-embedded, formalin-fixed tissue were used for classical diagnostic description as well as after picrosirius staining for evidencing the collagen structures. Confocal microscopy was used to evaluate unstained, freshly harvested samples, directly apposed with the mucosal side on the glass, using one-photon (reflectance confocal mode—RCM—for histological-like imaging) and 2-photon (second harmonic generation—SHG—for collagen visualisation) confocal microscopy. The results showed that, already at one month after the end of the inductions, both treatments were able to generate valuable modifications of the colon mucosa structures. Figure 3 displays the images of healthy colon mucosa obtained by classic histology and Figure 4 by 1- and 2-photon confocal microscopy. The figures document that, in healthy conditions, the colon mucosa had an orderly structure, with regular distribution and homogeneous shape of the crypts (Figure 3A and Figure 4A,C). This is in relation with the well-organised architecture of the collagen scaffold, as demonstrated by the SHG imaging (Figure 4B,D). The picrosirius staining also evidenced how the immune cells in the inter-cryptal spaces are in relation with the delicate collagen net that sustains and symmetrically spaces the crypts (Figure 3C,D).

The histology of the DSS-treated animal samples, in comparison with the healthy colon, demonstrated that colitis was present, confirmed by the diffused parvocellular infiltrate in the mucosa and submucosa, variation in size and shape of crypts, the occasional appearance little ulcers (Figure 3E,F), and enhancement of the collagen structure, evidenced by the picrosirius staining (Figure 3G,H). After AOM induction, the histology showed a lower degree of alterations than for DSS, with mild increase of immune infiltrate and some variability in the diameter of the crypts as well as widening of inter-cryptal distance. The picrosirius staining highlighted enhancement of the connective structure (Figure 3I–L).

The RCM imaging by 1-photon confocal microscopy also showed that the DSS induction produced variations of crypts in their shape (from round to prevalently ellipsoid appearance, with lozenge-like central lumen) and in their symmetric distribution with irregular inter-cryptal distance (Figure 4E,G). Similar changes, but at a lower extent, were evidenced also after AOM induction (particularly in the lower part of the colon), as well as enlarged inter-cryptal spaces (Figure 4I,K).

The modifications that both treatments induced in the collagen scaffold appeared more relevant when evaluated in SHG. In the healthy colon, the collagen scaffold appears as a continuous net of fibres organised to obtain a cribriform texture. This is supposed to allow elasticity to the structure and spaces enabling immune cell location and transit (Figure 4B,D). Round and regularly spaced holes indicate the places where the crypts are sited, symmetrically distributed and at an almost constant inter-cryptal distance between each other. This regular condition is altered after administration of DSS (Figure 4F,H) and AOM (Figure 4J,L). In both cases, the thickening and loosing of orderly architecture of the collagen scaffold is clearly visible, accompanied, at various extents, by asymmetry and distortion of the general structure, more evident after DSS-induced colitis.

**Figure 5:**
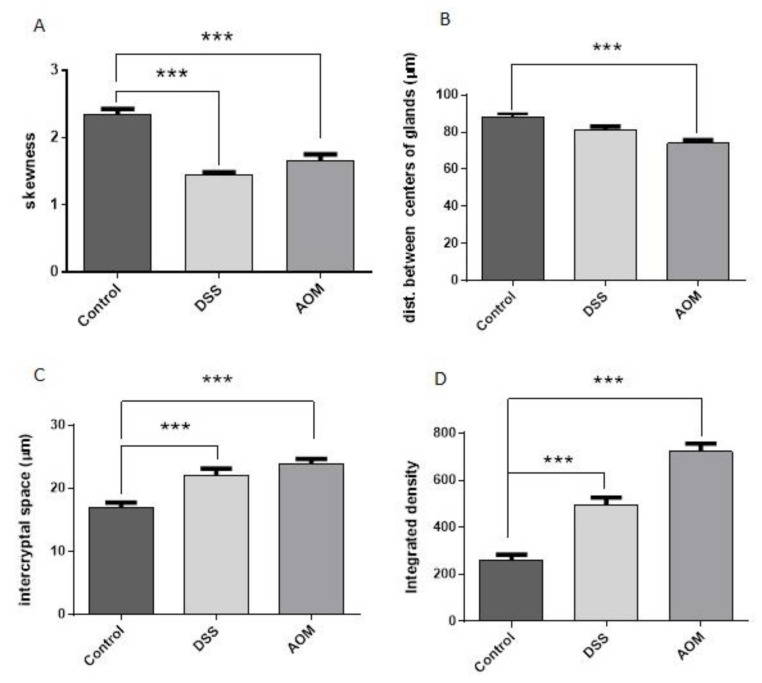
Evaluation of collagen distribution and colon tissue structures. In comparison to the control, the skewness variation (**A**) was significant in both DSS and AOM groups; the distance between centres of the crypts (**B**) was significant after AOM, while the inter-cryptal space wideness (**C**) was significant in both DSS- and AOM-treated groups. The integrated density (**D**) showed significant differences from the control and between the groups at *p* < 0.001. Significance by Kruskal–Wallis test: *** *p* < 0.001 in comparison to the untreated control.

All the cited parameters, i.e., increase of scaffold thickness and complexity (integrated density), divergence of symmetry and regularity of fibrous architecture (skewness), inter-cryptal distance, and distance between the centres of the crypts, were measured and evaluated (Figure 5). Shift towards increased inhomogeneity and irregularity of the system was indicated by skewness value reduction under both treatments (about −25% after AOM and −35% after DSS in comparison to the control) especially under DSS-induced inflammation. Variations of inter-cryptal spaces modifying the crypt symmetric distribution were also documented but, more importantly, the integrated density showed that the level of collagen deposition and assembling can differently characterise the conditions either of chronic inflammation or pro-carcinogenic microenvironment induced by DSS or AOM administration, respectively.

### 3.2. Selected Cytokine Production and Gene Expression in the Colon Mucosa 1 Month after DSS or AOM Inductions

In consideration that even though the two inducers are different molecules that act with different mechanisms, the effects on the colon mucosa evidenced by the microscopic evaluation (pro-fibrotic evolution, remodelling of the collagen scaffold, and presence of parvocellular immune infiltrate) were similar, we assumed inflammation would be the common factor in both conditions. Detection of IL-1α, IL-6, IFN-γ, and TGF-β1 levels by ELISA and gene expression of *Il1a, Il1b, Ifng,* and *Tgfb1* by RT-PCR was performed on samples of full colon mucosa. In consideration that the left colon is the most involved in both chronic colitis (ulcerative colitis) and tumours, we evaluated the right colon and the left colon mucosa separately to evidence possible differences of the immune response in the two parts of the colon under the two inductions.

#### 3.2.1. ELISA Results

**Figure 6:**
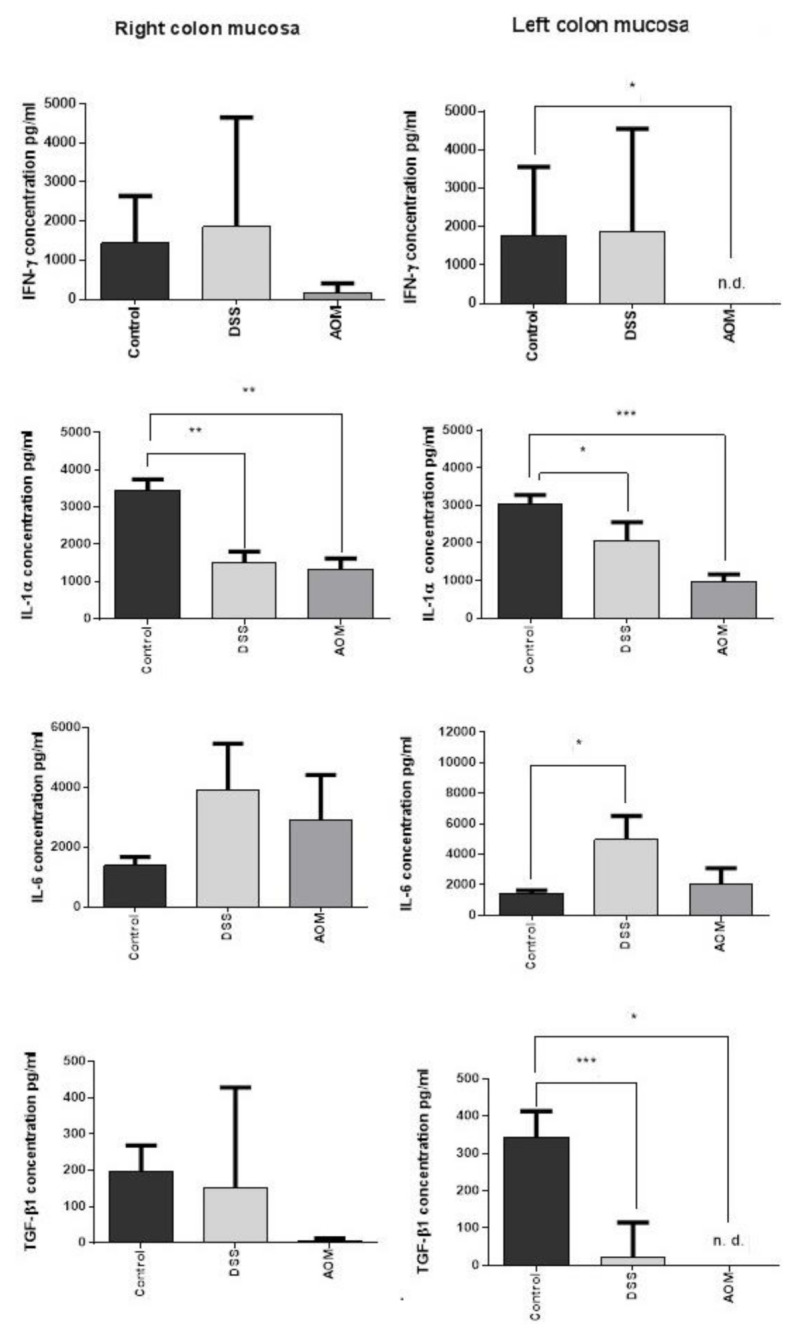
Cytokine production in the full colon mucosa of DSS- or AOM-induced rats at 1 month from the end of treatments. The production of all measured cytokines in the right and left colon mucosa showed similar patterns. The significant differences were found in the IL-1α production in both left and right colon between control and treated rats. Increase of IL-6 was particularly significant in the left colon mucosa after DSS colitis induction. Decrease of TGF-β1 was relevant after AOM in all colon mucosa and significant after both treatments in the left colon mucosa, the part of the colon known to be more affected by ulcerative colitis and cancer occurrence. IFN-γ production was slightly increased after DSS (not significant), whereas it was inhibited in the right colon mucosa and especially in the left colon mucosa (not at measurable levels) after AOM pro-carcinogenic treatment. ELISA values are expressed in pg/mL. Significance by Kruskal–Wallis test: * for *p* < 0.05, ** for *p* < 0.01, and *** for *p* < 0.001.

At the chosen time point, the production of cytokines evaluated by sandwich ELISA assay was similar in both the left and right colon mucosa after each type of induction (Figure 6). Interleukin-6 was increased by both inductions in comparison to the control (twofold higher than control after AOM and sixfold higher after DSS), especially in the left colon mucosa after the DSS administration. The production of the other cytokines instead resulted in being lower or not being modified in relation to the control levels after each type of treatment, with the exception of a slight increase of IFN-γ after DSS. One month after the end of AOM induction, IFN-γ production was decreased in the right colon mucosa and was at non-detectable levels in the left colon mucosa. In both parts of the colon, significant downregulation of IL-1α was also a common feature of both treatments (especially after AOM in the left colon, *p* < 0.001). The TGF-β1 production was significantly lower in the left colon mucosa than in the control mucosa. These results revealed an altered balance between regulatory and pro-inflammatory cytokines, still favourable to inflammation despite the generally reduced cytokine levels. This suggests the hypothesis of a phase in which the microenvironment attempts to recover and adapt after the acute response to the inducers. During this period, the progression to chronic inflammation may be sustained by the removal of an important regulator such as TGF-β1, with the effect of lowering the inflammatory threshold of the tissue. We suggest the existence of an “inflammatory threshold” that can be defined as the regulatory limit within which the inflammatory signals are tolerated without inducing pathological changes in a tissue. The increase of IL-6 in the DSS-treated group and, at a lesser extent after AOM, and altogether the persistence of IL-1α and IFN-γ at still valid levels in relation to TGF-β1, can cooperate to maintain the activity of the inflammation. This is particularly evident after DSS treatment and in the left colon, the target organ of colitis and cancer.

#### 3.2.2. RT-PCR Results

**Figure 7:**
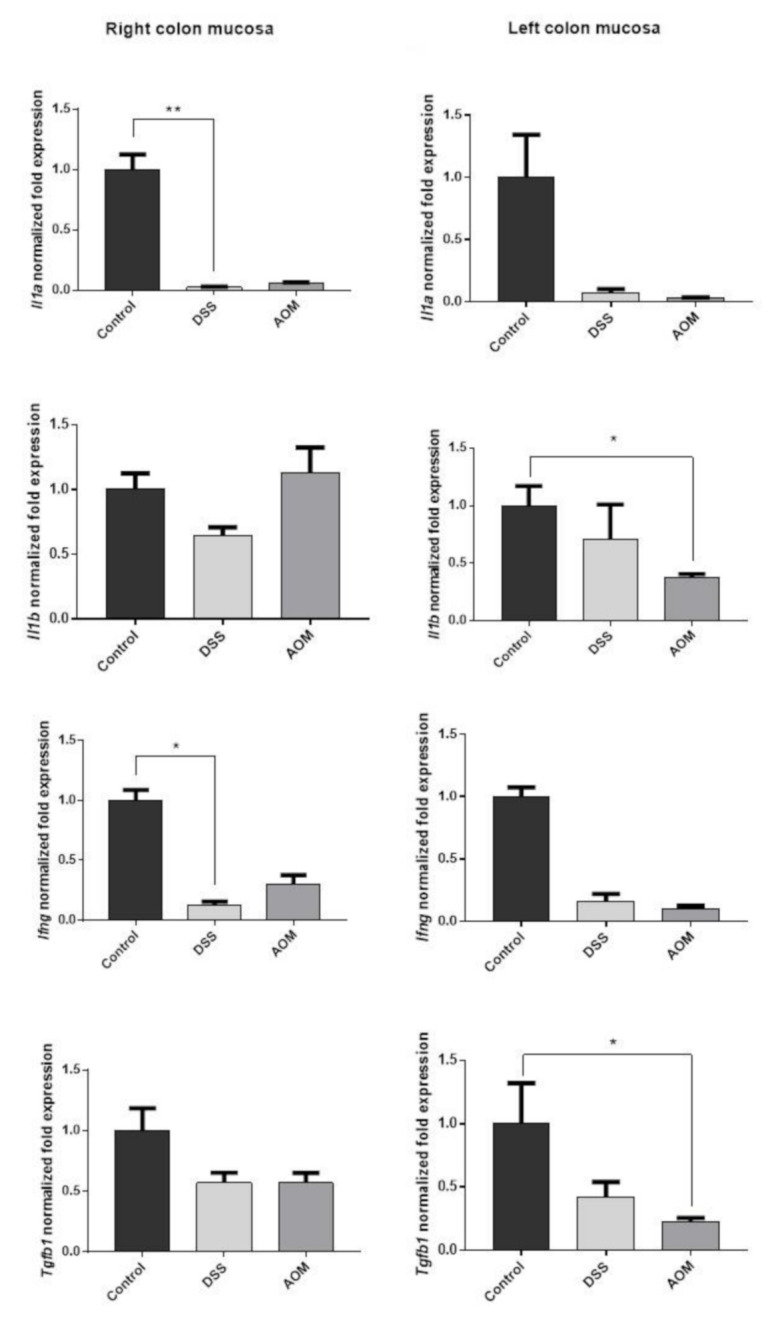
The expression of cytokines in the full colon mucosa of DSS- or AOM-induced rats at 1 month after the end of treatments. The expression of all measured genes in the induced rats, either after DSS or AOM, was reduced in comparison with control rats. The downregulation was present in all colons with exception of *Il1b* in the right colon, remaining at the control levels. Significant differences were found, particularly after AOM treatment in both left and right colon mucosa. Gene expression was evaluated according to expression of housekeeping gene *Ppia*. The expression stability of reference gene *Ppia* was confirmed using Normfinder, Bestkeeper, and RefFinder algorithms. Data were normalised to control groups. Significance by Kruskal–Wallis test: * for *p* < 0.05 and ** for *p* < 0.01.

The expression of *Il1a, Il1b, Ifng,* and *Tgfb1* was investigated in the right and left colon mucosa samples. At one month after the end of the induction with DSS or AOM, gene expression of pro-inflammatory cytokines resulted in generally being downregulated by both treatments in comparison to the expression in the mucosa of untreated rats (Figure 7). *Il1a* expression levels were much lower than the control in both the right and left colon. *Il1b* did not significantly change in the right colon, while it was significantly downregulated in the left colon after AOM (*p* < 0.05) but not after DSS induction. *Ifng* expression was reduced by both treatments in both parts of the colon.

About the regulatory cytokine TGF-β1, its gene expression was also significantly downregulated in the left colon, particularly after AOM treatment (*p* < 0.05). These data confirmed the presence of a downregulated immunological microenvironment during the evolution of chronic colitis and initial carcinogenesis processes at one month from the end of the inductions. They also indicated higher sensitivity of the left colon mucosa to the treatments. The downregulation was generally more significant in the AOM-induced rats.

#### 3.2.3. Regional and Systemic Immune Responses: Mesenteric Lymph Nodes and Spleen

**Figure 8:**
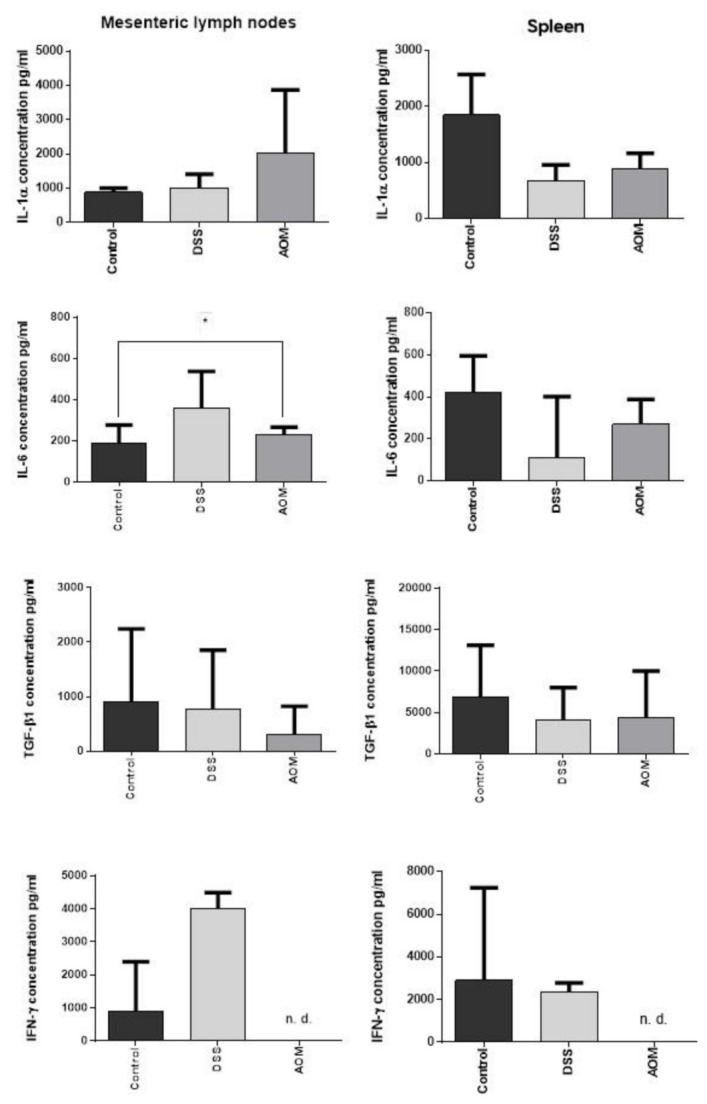
The production of IL-1α, IL-6, TGF-β1, and IFN-γ in mesenteric lymph nodes and spleen of DSS- or AOM-induced rats at 1 month from the end of treatments. The cytokine production in MLNs and spleen was indicative of the impact of DSS and AOM on immunity at systemic level. Signs of inflammatory activation were present in MLNs, even at a non-significant level, except for IL-6 after AOM (*p* < 0.05). Reduction (even though not reaching significance) of inflammatory cytokines and (at a lesser extent) TGF-β1 was observed in the spleen. IFN-γ resulted in being not detectable (n.d.) in AOM-treated groups. ELISA values are expressed in pg/mL. Significance was evaluated by Kruskal–Wallis test: * for *p* < 0.05.

To investigate a possibly consensual systemic immune response (regional and distant) to the treatments targeting the colon, we also performed a preliminary evaluation of the levels of IL-1α, IL-6, TGF-β1, and IFN-γ on mesenteric lymph nodes (MLN) and spleen samples. The MLN collect the lymph drained from the colon, and the spleen is a systemic immune organ. At the MLN level, DSS induction did not produce effects on the IL-1α and TGF-β1 cytokine levels. Even though the levels of IL-6 and IFN-γ appeared increased, no significance was found. The AOM induction, on one hand, produced increase of IL-6 with significance, and also a non-significant increase of IL-1α. On the other hand, decreased TGF-β1 and inhibited the IFN-γ production (Figure 8). This suggests inflammatory response at the regional level but of limited relevance. In the spleen, even though data did not reach significance, DSS and AOM decreased IL-1α and IL-6, and AOM, but not DSS, inhibited IFN-γ. The elevated level of TGF-β1, even in the untreated control, in comparison to the much lower production of the pro-inflammatory cytokines, suggests the spleen as a regulatory organ.

## 4. Discussion

The rat model in the present study was evidenced the possible changes produced in the local immunity and in the collagen scaffold by the induction of DSS chronic colitis or AOM carcinogenesis at one month from the end of the treatments. This period is intermediate between the acute immune response immediately at the term of the inductions and the finally established conditions of tumour or chronic colitis, which appear after three or more months from the induction [15,17]. This transition period is almost neglected and not described in the literature. Therefore, we compared the induced chronic colitis and the induced carcinogenesis to evidence whether, at this time point, possible similarities in immunological and structural changes may confirm inflammation as a progressive and central event in cancer development and remodelling of the collagen scaffold. In fact, remodelling of the tissue scaffold is considered a factor that can promote changes in the epithelial cell behaviour as well as in the local immunity [8,38,39]. Since both processes have the highest clinical relevance in the left colon, possible differences in the microenvironment of right and left mucosa were also considered.

In comparison to the control, similar changes in the evaluated cytokines under the two inductions were found in both parts of the colon, even though AOM appeared more active on the left colon mucosa. Notably, the findings revealed a downregulation of cytokine expression and production in comparison to the healthy mucosa, in particular of IL-1α and TGF-β1 by both inducers, while IFN-γ was especially affected by AOM administration, with relevant reduction in the left colon mucosa of the carcinogen-treated rats. Differently, the IL-6 cytokine increased in all colon mucosa under each of the treatments, particularly after DSS. This result is in accordance with the important role that IL-6 (involved in inflammation but also in tissue remodelling) has both in colitis and in tumour microenvironment development [40,41,42,43,44]. Even though the other inflammatory cytokines were downregulated, overall, they remained at higher levels in relation to the TGF-β1 regulatory cytokine that was decreased in the left colon by both inductions, especially AOM. TGF-β1 is a pleiotropic and regulatory cytokine [45]. It is considered as a double-edged sword in the carcinogenesis with a tumour-suppressing role in the early stages—e.g., in colon cancer development [46]. In later stages, it enhances tumour progression by contributing to the establishment of an immune suppressive microenvironment [47,48,49]. The TGF-β1 knockout animal models resulted in the elimination of advanced tumours compared to controls, suggesting importance of this cytokine in developed CRC [50,51]. Its reduction at one month after the carcinogenesis induction may underline one of the possible mechanisms favouring the establishment of the developing tumour in the colon. It was observed that AOM can negatively affect TGF-β1 and its pathway [52]. Interestingly, the cytokine production in both left and right colon mucosa was more affected after AOM than after DSS induction. After DSS, the inhibition was almost null in the right colon—generally less prone to colitis, than in the left colon, the target of colitis and even of sporadic or colitis-associated CRC. These differences may be secondary to differences in the local microbiome and its function in metabolising the inducers, especially the AOM that has a specific enterohepatic metabolism to be activated in its more toxic methylazoxymethanol [53,54,55,56,57].

The data about MLN and spleen cytokine variations indicate a possible immune activation at the regional (MLN) and systemic (spleen) levels. They suggest a pro-inflammatory response induced in the referent extra-colonic organ (regional lymph nodes), again sustained by IL-6 prevalence and apparently supported also by IFN-γ in the DSS treated group. On the contrary, non-detectable levels of IFN-γ were found after AOM induction, suggesting even a possible toxic effect on immune cells by the carcinogen. Differently, the spleen appeared to not be involved, indicating that the level of the pathologic process is still local. The relevant levels of TGF-β1 in the spleen, almost unchanged after the inductions, suggest a possible role in the maintenance of the systemic immune homeostasis. Splenic stroma-educated regulatory dendritic cells, for example, induce apoptosis of activated CD4 T cells via Fas ligand-enhanced IFN-γ and nitric oxide [57].

The described downregulation of the evaluated cytokines at one month from the end of the DSS and AOM inductions is a new insight that needs further investigations to understand the bases of these effects after the two types of induction. On the basis of these preliminary investigation results, we can hypothesise an attempt to recover homeostatic conditions after the acute effects of the treatments and/or an “exhaustion” of the local immunity. However, the direct toxicity of the two inducers (or their metabolic products) may also have a negative modulatory effect on immune cells, their viability, and their cytokine production [26,58,59]. Moreover, the responses to DSS or AOM treatment can differ between various rat and mouse strains, suggesting significant involvement of genetic background on the immune response [60,61]. Our observations also suggest the possible involvement of the microbiome in regulating the local immunity in the left or right colon, either due to changes of the microflora directly produced by the inducers or by different metabolic elaboration of the inducers, especially the carcinogen [12,62,63,64,65].

Very interesting and novel results were obtained from the microscopic analyses in multiphoton confocal microscopy using the SHG technique of imaging. Already at one month after both treatments, changes in the colon collagen structure were visible. Alteration of the collagen scaffold was already observed in other CRC-based studies using SHG microscopy for detection of changes in the structure of tissues [66,67,68]. Variation of symmetry as well as of collagen accumulation density in both conditions strongly highlight similar pathogenic mechanisms, i.e., response to inflammation and IL-6 activity. It is known from other studies that the immune activation in the colon can shape the collagen scaffold and that in the particular conditions of the tumour microenvironment, the tumour cells are able to set their own stroma to achieve tumour-promoting functions [69,70,71,72]. Moreover, the stiffening of a tissue due to the increase of the collagen accumulation and reorganisation can promote epithelial to mesenchymal transition and even affect the immune cell responses [69,73,74]. The processes of stromal remodelling that we observed in early period after the DSS and AOM inductions present different characteristics in symmetry of the structures and collagen deposition that can be evidenced and measured by SHG imaging. A relevant result is that the control, inflammation, and carcinogenesis groups showed significantly different integrated density (Figure 3), the values related to the signal obtained by the collagen deposited in the evaluated microscopic field. This early detection of collagen structure changes is very promising and can offer new possibilities to implement the pathological diagnosis as a new predictive marker, helping to discriminate between colon inflammation and cancer risk patients. Together with the evaluation of the local immunity, it could help in enhancing the possibilities of diagnosis, treatment, or prevision of these diseases [75,76].

The development of the tumour microenvironment is assumed to be strictly connected to its immune components and their modulation. At present, inflammation is indicated as one of the most important cancer hallmarks [77]. In its evolution, it is characterised by the paradoxical reversion of the original anti-tumour response into a chronic, tissue-remodelling and tumour-sustaining inflammation [1,6]. The establishment of these conditions are still under clarification and the progression of the immunological and biological events is generally described as a continuous process. According to our results, different levels of inflammation may occur in the transition between acute and chronic conditions. Apparently, the downregulation that we observed represents a discontinuity in the progression toward a fully established chronic inflammation. Nevertheless, it is the proportion between the levels of the various cytokines and the balance between pro-inflammatory and regulatory signals that may sustain a “latent” but still effective inflammation that is able to promptly restart under adequate stimulation (i.e., promoters). The situation described in this study documents this latent immune environment and supports its persistent activity as a smouldering inflammation, allowing the alterations in the collagen scaffold, already present in both chronic colitis and carcinogenesis conditions at the same time point [78]. Consequently, it is possible to suggest the existence of an “inflammatory threshold” that in the colon mucosa should be represented by the limit of immune regulation able to contain the immune activation due to the presence of the microflora without damaging inflammation. In case of reduction of the inflammatory threshold, even mild inflammatory signals can actively play and efficiently prepare the progression of the disease [79].

## 5. Conclusions

The observation of the immune activity and collagen scaffold changes at one month after the end of induction either with DSS or AOM has evidenced the possibility of transitory periods in which remodelling of tissue is assisted by a “remodelled” immune response. It can even be a downregulated response but one that is still able to maintain the inflammatory background, despite the apparent recovery from more intense inflammatory conditions. This is a proper example of smouldering inflammation that the changes in the collagen scaffold, first here described, can help to identify. These results provide support to a model of very plastic tumour microenvironment development, modulated and sustained by the variation of the mucosal inflammatory threshold, and propose new possible pathological markers for better distinguishing chronic and pre-neoplastic inflammation in the colon mucosa.

## Figures and Tables

**Table 1:** List of primers used in RT-PCR analysis.

Gene	Forward Primer	Reverse Primer
*Il1a*	AAGACAAGCCTGTGTTGCTGAAGG	TCCCAGAAGAAAATGAGGTCGGTC
*Il1b*	CACCTCTCAAGCAGAGCACAG	GGGTTCCATGGTGAAGTCAAC
*Ifng*	ACTGGCAAAAGGACGGTAAC	ATCAGGTGCGATTCGATGAC
*Tgfb1*	TGAGTGGCTGTCTTTTGACG	TCTGTGGAGCTGAAGCAGTA
*Ppia*	TACAGGTCCTGGCATCTTGT	AGTTGTCCACAGTCGGAGAT

## Data Availability

The data presented in this study are available on request from the corresponding author. The data are not publicly available due to privacy reasons.

## Supplementary Materials

The following are available online at https://www.mdpi.com/article/10.3390/cancers13102463/s1, Figure S1: Representative melting curves for the genes used in the study, Table S1: Values of ELISA, PCR and imaging analysis statistical evaluations.
