# Supplementary material for: Local Immune Changes in Early Stages of Inflammation and Carcinogenesis Correlate with the Collagen Scaffold Changes of the Colon Mucosa
Source: Cancers (Basel). 2021 May 18;13(10):2463. doi: 10.3390/cancers13102463 (PMC8158480; doi:10.3390/cancers13102463)
Supplement: Supplementary file 1 [file cancers-13-02463-s001.zip › cancers-1186695-supplementary.pdf]

cancers

MDPI

Supplementary Materials: Local Immune Changes in Early Stages of Inflammation and Carcinogenesis Correlate with the Collagen Scaffold Changes of the Colon Mucosa

Fabián Čaja, Dmitry Stakheev, Oleksandr Chernyavskiy, Lucie Kubinová, Jiří Křížan, Jiří Dvořák, Pavel Rossmann, Renata Štěpánková, Peter Makovický, Pavol Makovický, Veronika Vymetalková, Pavel Souček, Pavel Vodička, L'udmila Vodičková, Miroslav Levý and Luca E. Vannucci

Figure S1. Representative melting curves for the genes used in the study.The description of the procedure is reported at paragraph 2.3

Ppia

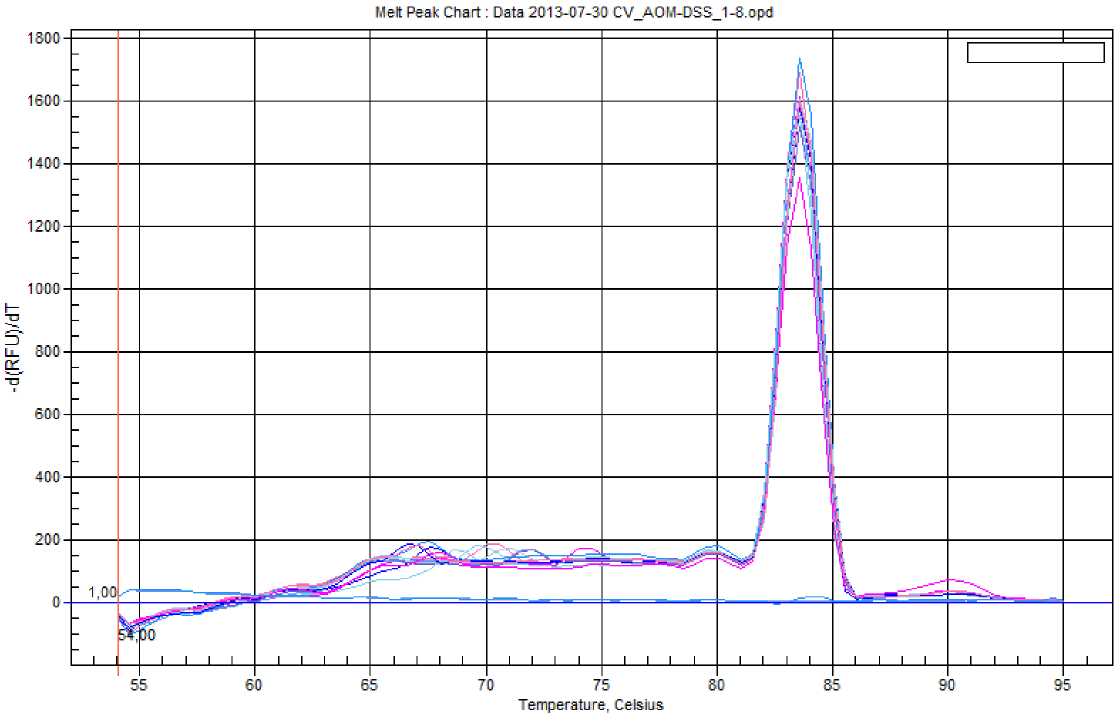

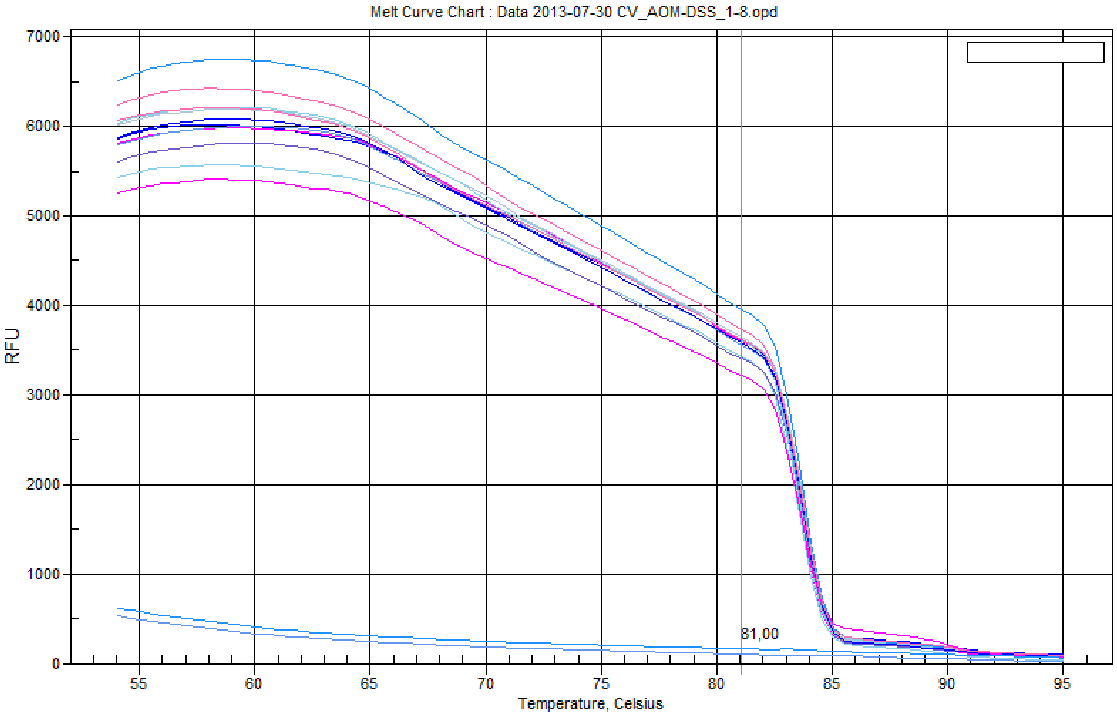
Melt Curve Chart : Data 2013-07-30 CV_AOM-DSS_1-8.opd
RFU
7000
6000
5000
4000
3000
2000
1000
0
81,00
55
60
65
70
75
80
85
90
95
Temperature, Celsius

Il1a

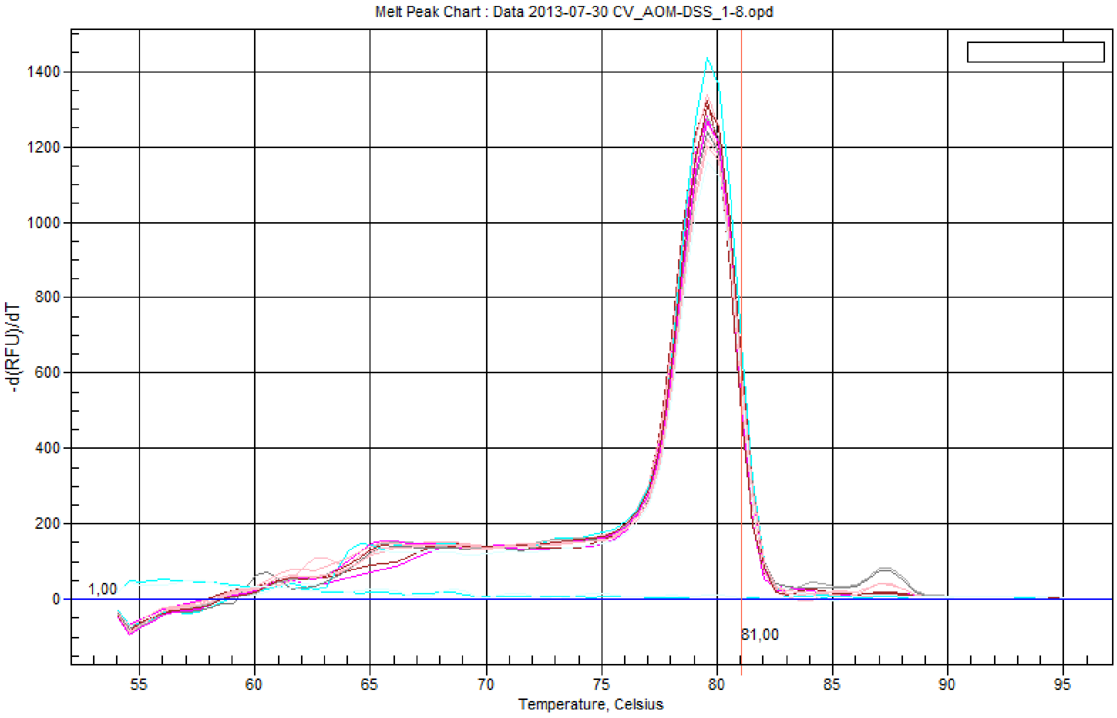
Melt Peak Chart : Data 2013-07-30 CV_AOM-DSS_1-8.opd
-d(RFU)/dT
1400
1200
1000
800
600
400
200
0
1,00
81,00
55
60
65
70
75
80
85
90
95
Temperature, Celsius

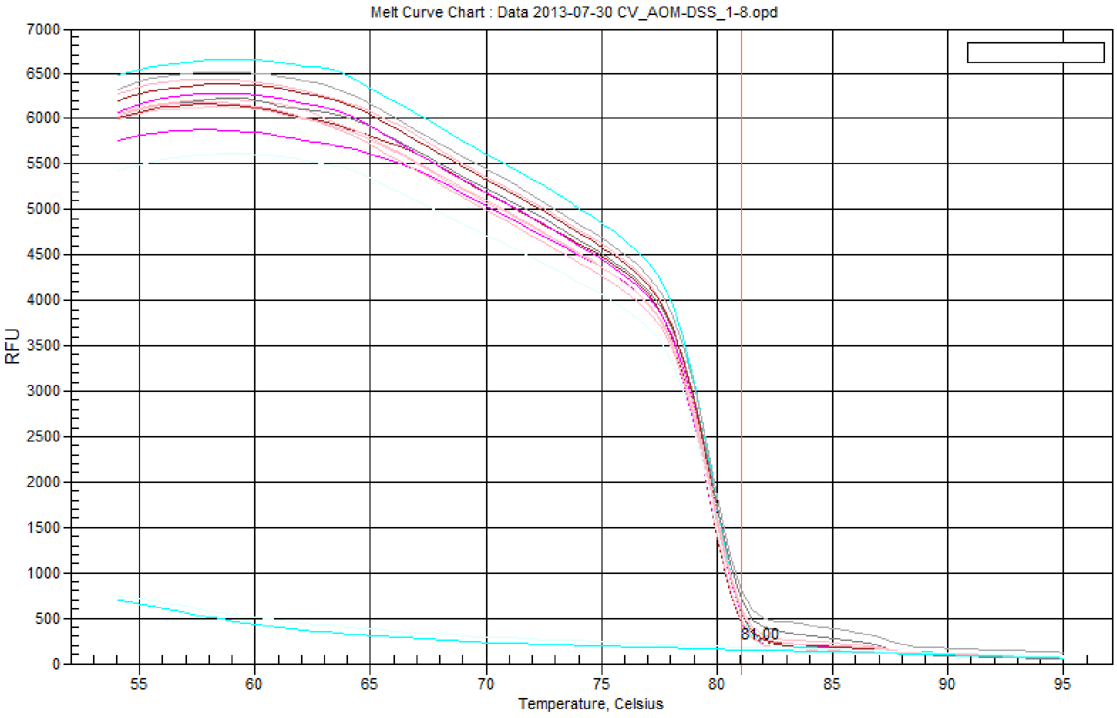
Melt Curve Chart : Data 2013-07-30 CV_AOM-DSS_1-8.opd
RFU
Temperature, Celsius
81,00

Il1b

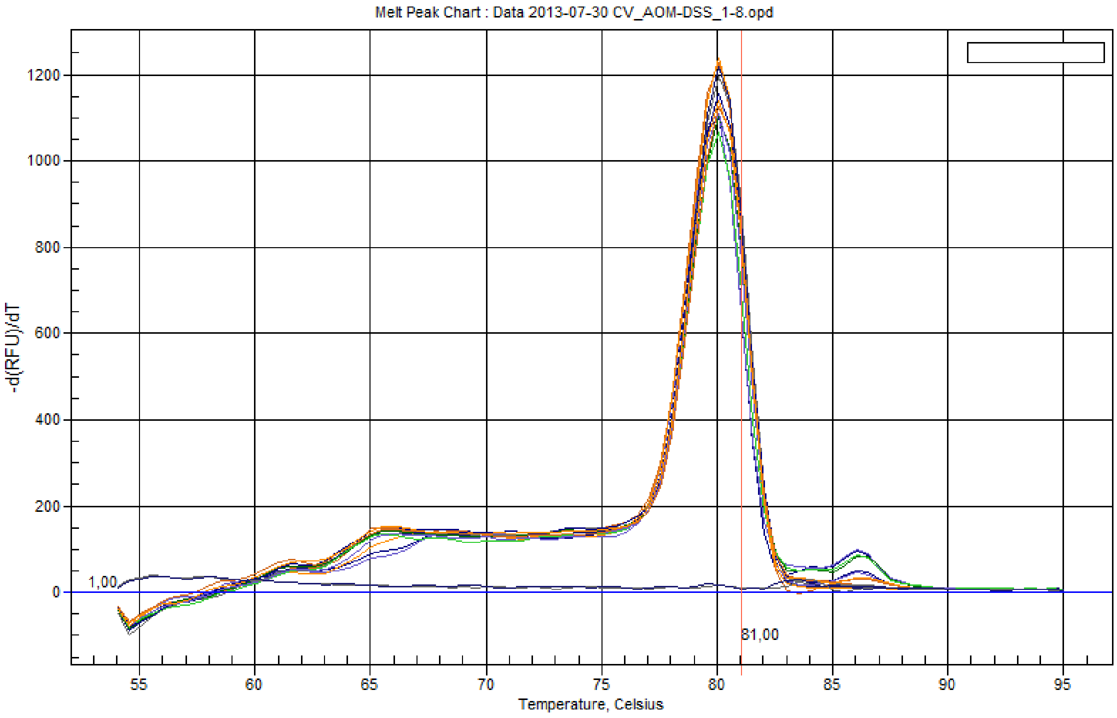
Melt Peak Chart : Data 2013-07-30 CV_AOM-DSS_1-8.opd
-d(RFU)/dT
Temperature, Celsius
1,00
81,00

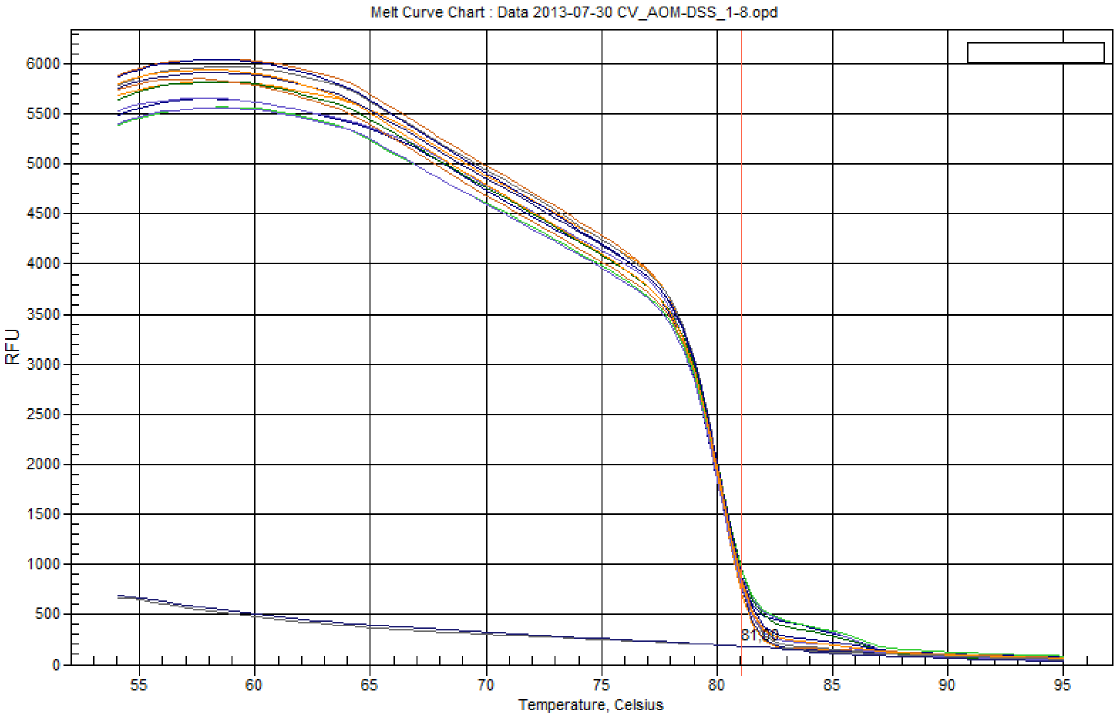
Melt Curve Chart : Data 2013-07-30 CV_AOM-DSS_1-8.opd
RFU
6000
5500
5000
4500
4000
3500
3000
2500
2000
1500
1000
500
0
81,00
55
60
65
70
75
80
85
90
95
Temperature, Celsius

Ifng

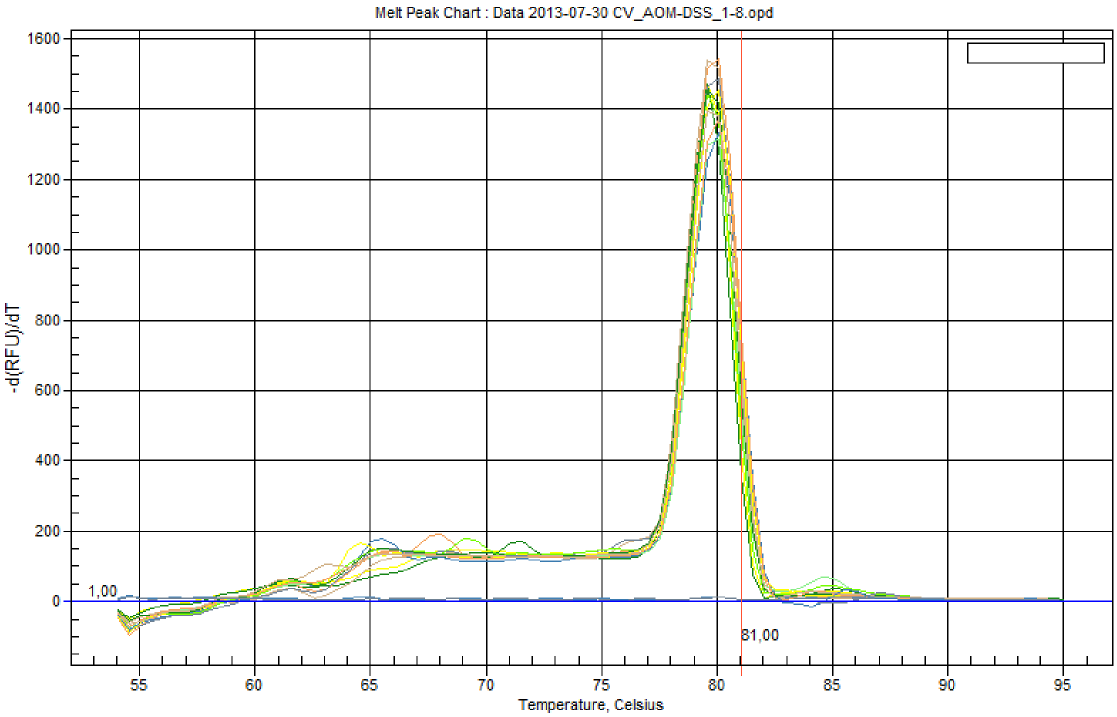
Melt Peak Chart : Data 2013-07-30 CV_AOM-DSS_1-8.opd
-d(RFU)/dT
1600
1400
1200
1000
800
600
400
200
0
1,00
81,00
55
60
65
70
75
80
85
90
95
Temperature, Celsius

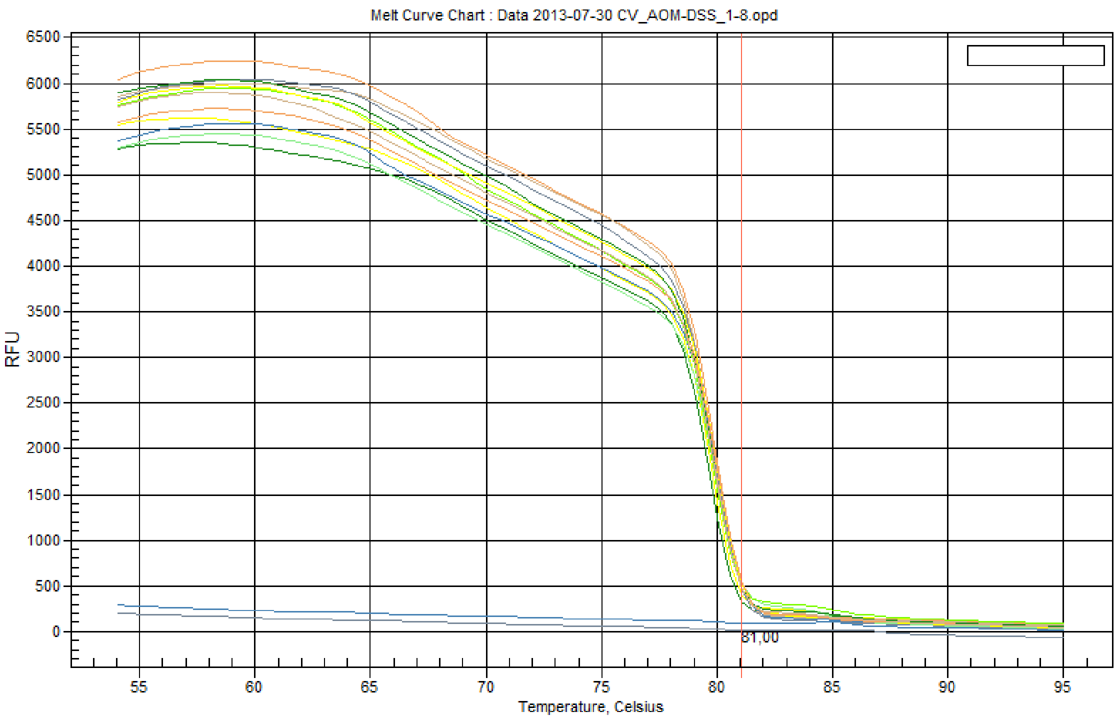
Melt Curve Chart : Data 2013-07-30 CV_AOM-DSS_1-8.opd
RFU
6500
6000
5500
5000
4500
4000
3500
3000
2500
2000
1500
1000
500
0
81,00
55
60
65
70
75
80
85
90
95
Temperature, Celsius

Tgfb1

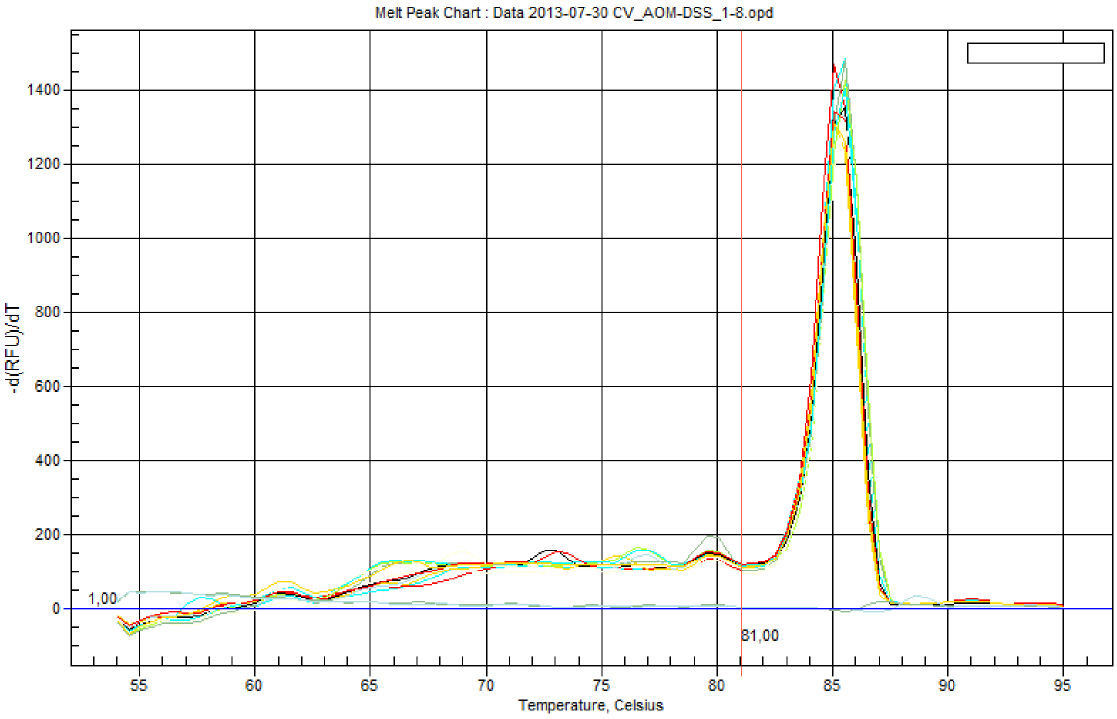
Melt Peak Chart : Data 2013-07-30 CV_AOM-DSS_1-8.opd
-d(RFU)/dT
1400
1200
1000
800
600
400
200
0
1,00
81,00
55
60
65
70
75
80
85
90
95
Temperature, Celsius

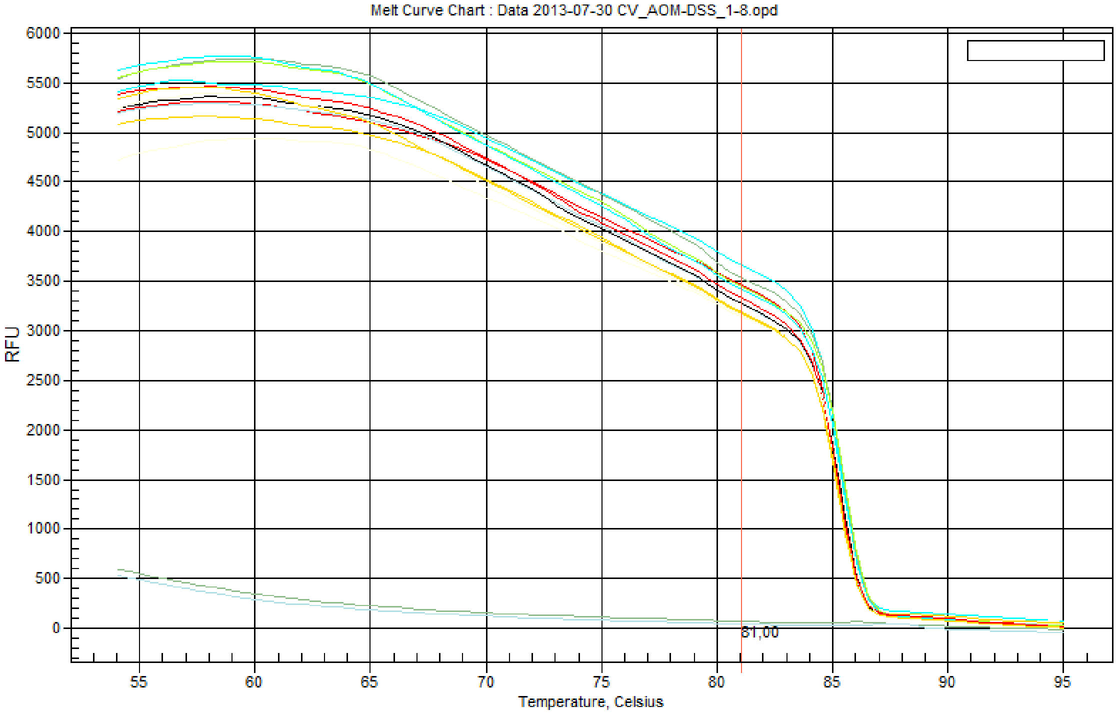

Table S1. Values of ELISA, PCR and imaging analysis statistical evaluations.

Differences in distributed variables between groups were compared by one-way ANOVA and subsequent Kruskal–Wallis test using GraphPad Prism™. Statistical significance was considered for $p < 0.05$

ELISA

p-value shown as comparison of treated rats with controls

Protein	Tissue	Treatment	p-value	Mean	S.E.M.	N
IL-1α		control	-	3047,0	229,2	47

	Left colon mucosa	DSS	0,0132	2068,0	485,7	20
		AOM	<0,0001	971,8	194,1	15
	Right colon mucosa	control	-	3438,0	299,4	31
		DSS	0,0013	1501,0	300,4	15
		AOM	0,0013	1328,0	288,6	11
	MLN	control	-	878,3	123,0	26
		DSS	>0,9999	1010,0	397,9	6
		AOM	0,958	2021,0	1854,0	4
	Spleen	control	-	1846,0	719,8	26
		DSS	0,4471	678,3	280,4	8
		AOM	>0,9999	890,3	270,5	6
IL-6	Left colon mucosa	control	-	1405,0	228,1	55
		DSS	0,0223	4945,0	1567,0	19
		AOM	>0,9999	2030,0	1051,0	9
	Right colon mucosa	control	-	1387,0	291,6	29
		DSS	0,624	3908,0	1561,0	17
		AOM	0,5336	2895,0	1527,0	6
	MLN	control	-	188,5	88,4	30
		DSS	0,1074	357,9	179,2	6
		AOM	0,0441	230,4	36,0	4
	Spleen	control	-	419,4	175,8	26
		DSS	>0,9999	110,1	110,1	7
		AOM	0,3964	270,9	117,1	4
TGF-β1	Left colon mucosa	control	-	342,6	70,6	67
		DSS	0,0005	21,9	17,9	27
		AOM	0,0157	0,0	0,0	9
	Right colon mucosa	control	-	196,2	72,9	60
		DSS	>0,9999	152,6	65,1	18
		AOM	>0,9999	6,3	6,3	8
	MLN	control	-	909,9	213,6	39
		DSS	>0,9999	772,4	441,7	6
		AOM	>0,9999	305,1	259,2	4
	Spleen	control	-	6818,0	914,3	47
		DSS	0,3019	4104,0	971,5	16
		AOM	0,5115	4340,0	2305,0	6
IFN-γ	Left colon mucosa	control	-	1758,0	252,7	51
		DSS	0,3592	1878,0	715,5	14
		AOM	0,0372	0,0	0,0	3
	Right colon mucosa	control	-	1438,0	236,3	26
		DSS	0,9237	1856,0	988,9	8
		AOM	0,1589	150,0	150,0	3

	MLN	control	-	874,3	324,7	22
		DSS	0,1085	4013,0	337,5	2
		AOM	0,5726	0,0	0,0	3
	Spleen	control	-	2899,0	972,4	20
		DSS	>0,9999	2344,0	312,5	2
		AOM	0,8326	0,0	0,0	2

PCR

p-value shown as comparison of treated rats with controls

Gene	Tissue	Treatment	p-value	Mean	S.E.M.	N
Il1a	Left colon mucosa	control	-	11,950	4,0760	4
		DSS	0,2184	0,851	0,3740	8
		AOM	0,1361	0,345	0,0850	9
	Right colon mucosa	control	-	11,070	1,3810	3
		DSS	0,0053	0,257	0,0663	9
		AOM	0,1967	0,598	0,1265	8
Il1b	Left colon mucosa	control	-	1,093	0,1881	4
		DSS	0,2085	0,771	0,3318	6
		AOM	0,0458	0,410	0,0351	7
	Right colon mucosa	control	-	0,976	0,1223	3
		DSS	0,3169	0,576	0,0605	3
		AOM	>0,9999	1,104	0,1937	4
Ifng	Left colon mucosa	control	-	1,847	0,1390	2
		DSS	0,1506	0,296	0,1084	7
		AOM	0,0725	0,183	0,0481	8
	Right colon mucosa	control	-	1,896	0,1658	2
		DSS	0,0193	0,236	0,0562	8
		AOM	0,3099	0,574	0,1370	9
Tgfb1	Left colon mucosa	control	-	2,657	0,8547	4
		DSS	0,536	1,126	0,3089	7
		AOM	0,0375	0,608	0,0749	7
	Right colon mucosa	control	-	1,688	0,3139	3
		DSS	0,1307	0,959	0,1438	8
		AOM	0,1276	0,960	0,1379	9

IMAGING

p-value shown as comparison of treated rats with controls

**Parameter**	**Treatment**	**p-value**	**Mean**	**S.E.M.**	**N**
skewness	control	-	2,342	0,0868	107
	DSS	<0,0001	1,435	0,0499	104
	AOM	<0,0001	1,663	0,0894	72
dist. between centers of the glands	control	-	87,940	2,0070	136
	DSS	0,2742	81,370	1,6910	126
	AOM	<0,0001	73,900	1,9650	77
intercryptal space	control	-	16,930	0,8036	126
	DSS	0,0004	22,010	1,1320	81
	AOM	<0,0001	23,860	0,7971	216
integrated density	control	-	261,500	23,1800	130
	DSS	<0,0001	492,000	34,7800	114
	AOM	<0,0001	722,300	33,7100	216
